# Repetitive Transcranial Magnetic Stimulation in Youth With Treatment Resistant Major Depression

**DOI:** 10.3389/fpsyt.2019.00170

**Published:** 2019-03-29

**Authors:** Frank P. MacMaster, Paul E. Croarkin, T. Christopher Wilkes, Quinn McLellan, Lisa Marie Langevin, Natalia Jaworska, Rose M. Swansburg, Yamile Jasaui, Ephrem Zewdie, Patrick Ciechanski, Adam Kirton

**Affiliations:** ^1^Departments of Pediatrics and Psychiatry, Cumming School of Medicine, University of Calgary, Calgary, AB, Canada; ^2^Strategic Clinical Network for Addictions and Mental Health, Alberta Health Services, Calgary, AB, Canada; ^3^Department of Psychiatry and Psychology, Mayo Clinic, Rochester, NY, United States; ^4^Institute of Mental Health Research, University of Ottawa, Ottawa, ON, Canada; ^5^Departments of Pediatrics and Clinical Neurosciences, Cumming School of Medicine, University of Calgary, Calgary, AB, Canada

**Keywords:** adolescent, depression, transcrancial magnetic stimulation (TMS), dorsolateral prefrontal cortex (DLPFC), brain stimulation

## Abstract

**Background:** Major depressive disorder (MDD) is common in youth and treatment options are limited. We evaluated the effectiveness and safety of repetitive transcranial magnetic stimulation (rTMS) in adolescents and transitional aged youth with treatment resistant MDD.

**Methods:** Thirty-two outpatients with moderate to severe, treatment-resistant MDD, aged 13–21 years underwent a three-week, open-label, single center trial of rTMS (ClinicalTrials.gov identifier NCT01731678). rTMS was applied to the left dorsolateral prefrontal cortex (DLPFC) using neuronavigation and administered for 15 consecutive week days (120% rest motor threshold; 40 pulses over 4 s [10 Hz]; inter-train interval, 26 s; 75 trains; 3,000 pulses). The primary outcome measure was change in the Hamilton Depression Rating Scale (Ham-D). Treatment response was defined as a >50% reduction in Ham-D scores. Safety and tolerability were also examined.

**Results:** rTMS was effective in reducing MDD symptom severity (*t* = 8.94, *df* = 31, *p* < 0.00001). We observed 18 (56%) responders (≥ 50% reduction in Ham-D score) and 14 non-responders to rTMS. Fourteen subjects (44%) achieved remission (Ham-D score ≤ 7 post-rTMS). There were no serious adverse events (i.e., seizures). Mild to moderate, self-limiting headaches (19%) and mild neck pain (16%) were reported. Participants ranked rTMS as highly tolerable. The retention rate was 91% and compliance rate (completing all study events) was 99%.

**Conclusions:** Our single center, open trial suggests that rTMS is a safe and effective treatment for youth with treatment resistant MDD. Larger randomized controlled trials are needed.

**Clinical Trial Registration:** www.ClinicalTrials.gov, identifier: NCT01731678

## Introduction

Treatment options for major depressive disorder (MDD) in youth are limited, with concerns over efficacy (1) and safety (2–4). Evidence suggests 30–50% of adolescents and young adults with MDD are treatment-resistant, leading to lifelong consequences (5). Consequently, new interventions for treatment-resistant MDD in youth are needed (6). One potential treatment is repetitive transcranial magnetic stimulation (rTMS) (7). Prior research demonstrates that rTMS targeting the left dorsolateral prefrontal cortex (DLPFC) in adults is well-tolerated, safe, and effective for MDD (8–12). Prior studies (case series and reports) of rTMS in MDD patients below the age of 22 are limited but encouraging, but substantial knowledge gaps remain (13). With this in mind, we conducted a trial of LDLPFC rTMS in youth with treatment resistant MDD. We hypothesized that rTMS would reduce depressive symptom severity in youth with MDD.

## Materials and Methods

This study was approved by the Conjoint Health Research Ethics Board (CHREB) for the University of Calgary.

### Participants

Participants were recruited from the community and clinics in our area. Inclusion criteria were: (1) age 12–22 years, (2) diagnosis of MDD, based on an interview with the Kiddie Schedule for Affective Disorders and Schizophrenia Present and Lifetime version (K-SADS-PL) (14) with a symptom severity of 40 or greater on the Children's Depression Rating Scale Revised (CDRS-R) (15), (3) history of failing to respond to at least one selective serotonin reuptake inhibitors (SSRI) trial (minimum 8-week treatment at an adequate dose; retrospectively determined on interview), and (4) ability to provide informed consent. Psychotropic medications were allowed if the dose has been stable for 6 weeks with adequate compliance, and with a commitment to not change medication/dosage during the trial period, unless deemed medically necessary. Exclusion criteria included: (1) previous seizures or epilepsy, (2) hypertension, (3) previous neurological or psychiatric diagnoses (specifically—bipolar disorder or psychosis) (4) pregnancy, (5) implanted metal or medical device, or (6) left-handedness.

### Assessments

Participants were assessed at baseline, weekly during rTMS, and at the completion of treatment. Core clinical assessments were designed to confirm diagnosis (baseline), define symptoms, and assess treatment response. The K-SADS-PL (14), was used to assess both present and lifetime psychiatric symptomatology. The CDRS-R (15) was administered at baseline and treatment completion to quantify moderate to severe depressive symptom severity in participants. The Hamilton Depression Rating Scale (Ham-D) (16) and Beck Depression Inventory (BDI) (17), were used to assess depressive symptoms at baseline, the end of each week for safety monitoring, and after treatment completion. The Hamilton Anxiety Rating Scale (Ham-A) (18), to assess anxiety levels, was also administered at baseline and after treatment completion. We endeavored to complete assessments within 2 weeks of initiation and termination of rTMS.

The primary outcome measure of treatment response was defined as a > 50% reduction in Ham-D scores from baseline to end-point. A secondary exploratory measure of clinically meaningful response was also preset at 30% based on American College of Neuropsychopharmacology (ACNP) recommendations (19). We also examined predefined criteria for remission (Ham-D score ≤ 7 post-rTMS) and partial remission (Ham-D score ≤ 10 post-rTMS and a ≥ 50% reduction in Ham-D score).

### rTMS

TMS was performed at the Alberta Children's Hospital. The TMS intervention utilized a Magstim SuperRapid^2^, air-cooled 90 mm figure-of-8-coil (Magstim, Wales UK). Using a neuronavigation system (Brainsight2, Rogue Research, Montreal), the TMS coil was monitored in real time and co-registered with the individual's structural MRI. Imaging was performed on a 3T GE MR750w scanner using a 24-channel head coil. Anatomical scan parameters were: Axial T1-w 3D BRAVO with TR = 8,204 ms, TE = 3.168 ms, flip angle = 10 degrees, 226 slices with 0.8 mm thickness, 300 × 300 matrix.

On the first day, motor evoked potentials (MEP) were recorded to determine the resting motor threshold (RMT) in the standard manner (20). To initially locate the DLPFC target site, the 5-cm rule was applied, in which the scalp position 5 cm anterior to the hotspot along a line to the nasion was marked (21–24). Neuronavigation confirmed accurate DLPFC (adjusted if needed) targeting. The TMS coil was subsequently placed tangential to the scalp, and angled at 45 degrees to the midline and fixed over the target using a mechanical arm. The target was marked on the neuronavigation system, allowing real time targeting and accurate session to session reliability.

rTMS was applied at 10 Hz. Each train consisted of 40 suprathreshold (120% RMT) pulses over 4 s with an inter-train interval of 26 s. Treatment sessions lasted 37.5 min (75 trains/3,000 pulses), and occurred at the same time of day on every week day for a period of 3 weeks (15 days total). During TMS, only passive activities were allowed (i.e., watching movies or TV, listening to music). Three weeks of treatment was selected based on existing rTMS evidence in adult MDD and our rTMS experience in pediatric stroke (25, 26). Participants were monitored for adverse events and tolerability using a Pediatric TMS Safety and Tolerability Measure (27) on days 1, 6, and 11. Items were self-rated.

### Statistics

For the primary outcome, we hypothesized that rTMS would result in a significant reduction in depression symptom severity as measured by the Ham-D. We used a paired *t*-test comparing baseline to post-rTMS with *p* < 0.005. We increased the threshold in keeping with recommendations designed to improve reproducibility (28). For exploratory measures (i.e., CDRS, BDI, Ham-A) we used a paired *t*-test comparing baseline to post-rTMS with *p* < 0.01 to correct for multiple comparisons (Bonferroni). Analyses were performed with IBM SPSS (version 24; New York, USA).

## Results

**Figure 1:**
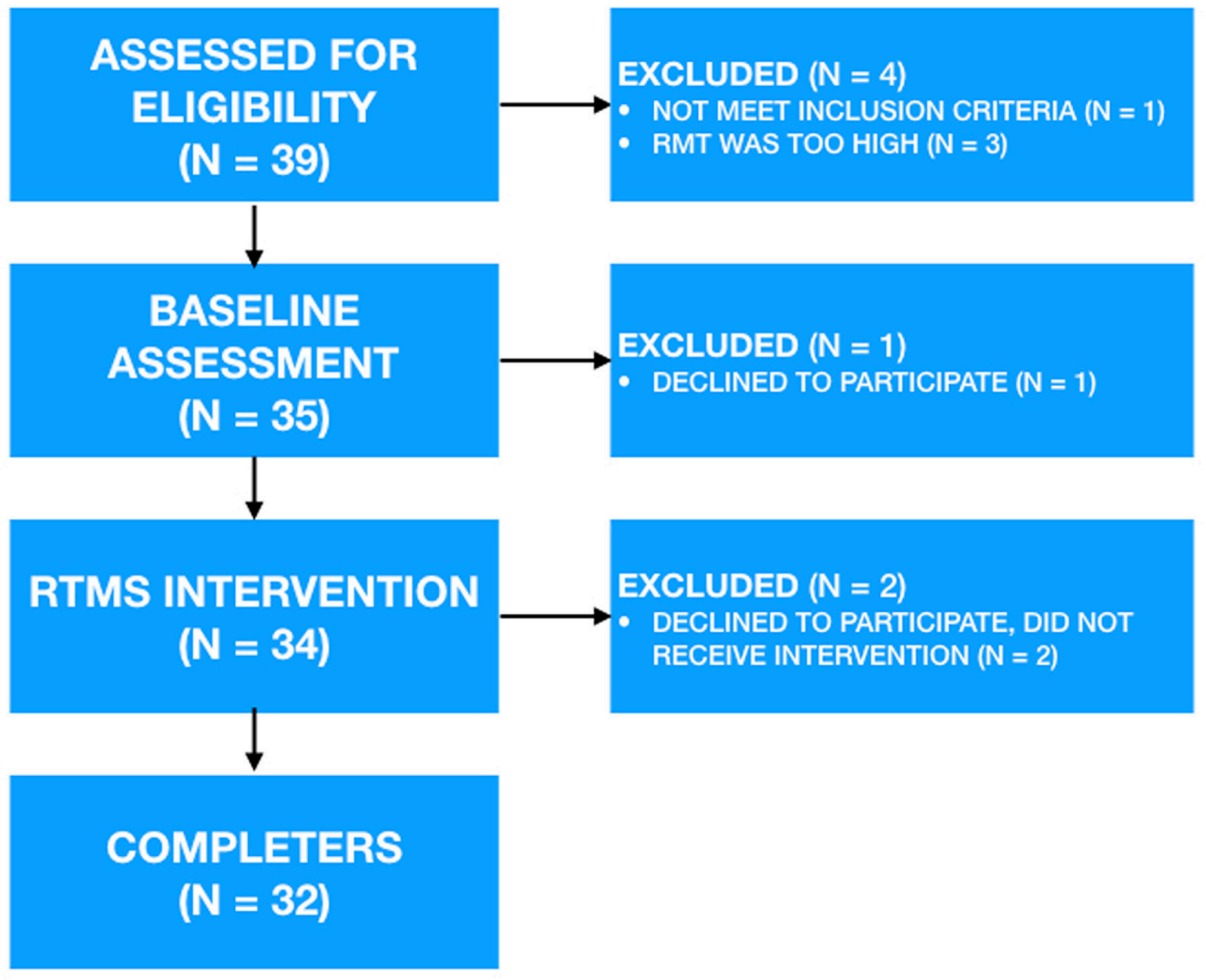
Basic study design. rTMS, repetitive transcranial magnetic stimulation; RMT, resting motor threshold.

Thirty-nine participants were recruited (Figure 1). Four were excluded due to high motor threshold precluding comfortable rTMS (3) and left-handedness (1). One participant withdrew during the baseline assessment. Two withdrew just prior to the rTMS intervention starting, due to the social aspects of the treatment (i.e., coming into a busy hospital). None reported withdrawal due to the rTMS. The sample consisted of 32 participants (46.9% female; see Table 1). At baseline, 5 were rated by the Ham-D as having mild depression, 9 as moderate, 4 as severe, and 14 as very severe. All participants had at least 2 failed medication trials. During the study, ten (31.25%) were medication free. The remainder reported medications including SSRIs (*n* = 14), norepinephrine and dopamine reuptake inhibitors (NDRI = 7), serotonin and norepinephrine reuptake inhibitors (SNRIs = 4), atypical antipsychotics (*n* = 3), benzodiazepines (*N* = 1), guanfacine (*N* = 1), lithium (*N* = 1), opioid analgesic (*N* = 1), antibiotic (*N* = 1), tetracyclic antidepressant (*N* = 1), and serotonin antagonist and reuptake inhibitors (SARI = 1). No participant changed their medication or dosage during the trial. Reported comorbid diagnosis in 28 (88%) participants included generalized anxiety disorder (GAD = 24), social phobia (*n* = 10), panic disorder (*n* = 8), attention deficit hyperactivity disorder (ADHD = 4), social anxiety disorder (*N* = 3), Asperger's (*N* = 2), post-traumatic stress disorder (PTSD = 2), specific phobia (*N* = 2), separation anxiety disorder, conduct disorder (*N* = 1), and bulimia (*N* = 1), and Ehler-Danlos syndrome (*N* = 1). Time from baseline assessment to the initiation of rTMS was 9.71 ± 14.93 days, and time between the end of rTMS and the final assessment was 3.45 ± 7.57 days.

**Table 1:** Clinical characteristics of 32 youth with treatment resistant depression treated with rTMS.

**#**	Age^*^	**Sex**	**Duration of illness**	**Ham-D**		**CDRS**		**BDI**		**Ham-A**	
				**Baseline**	**End**	**Baseline**	**End**	**Baseline**	**End**	**Baseline**	**End**
**MEAN** ± **STANDARD DEVIATION OVERALL**											
–	17.57 ± 1.98	–	3.85 ± 1.71	20.25 ± 6.37	9.38 ± 5.44	73.36 ± 9.07	59.72 ± 10.23	30.09 ± 9.32	16.19 ± 12.18	21.94 ± 8.18	10.84 ± 7.55
**INDIVIDUAL DATA**											
1	≥18	M	2.57	29	7	85	62	41	11	38	11
2	≥18	F	5.86	23	19	85	74	34	38	33	18
3	≥18	M	2.24	23	6	74	56	31	5	21	9
4	≥18	M	1.39	25	12	84	68	29	26	14	9
5	≥18	F	5.41	31	7	85	48	40	4	34	4
6	16–17	F	5.38	28	22	85	85	41	51	33	24
7	14–15	M	3.84	17	5	81	44	37	4	22	2
8	16–17	F	1.18	10	9	67	56	17	2	5	3
9	13≥	F	3.62	16	11	69	63	35	29	18	17
10	≥18	M	3.69	14	11	77	64	29	23	19	3
11	≥18	M	2.33	19	8	57	51	21	11	26	6
12	16–17	M	4.66	13	0	62	40	9	9	9	2
13	16–17	F	5.40	12	14	73	64	40	27	22	14
14	16–17	F	3.65	14	9	64	57	37	8	30	14
15	13≥	M	8.23	23	6	66	73	18	3	20	17
16	16–17	F	4.78	15	9	80	69	42	24	18	12
17	≥18	F	3.01	24	22	81	69	42	33	29	28
18	16–17	M	1.26	30	22	85	80	24	28	21	21
19	16–17	F	5.46	19	5	71	55	22	3	25	7
20	14–15	F	4.64	23	5	81	62	24	1	24	9
21	16–17	F	4.59	18	10	65	57	37	23	18	4
22	14–15	M	2.91	18	7	67	63	36	18	25	23
23	16–17	F	5.86	12	7	53	48	11	2	6	3
24	13≥	M	0.83	19	5	71	51	26	6	17	9
25	≥18	M	5.11	32	8	85	53	39	11	23	8
26	16–17	M	1.34	28	12	72	58	37	18	35	28
27	≥18	M	4.66	14	5	81	56	27	14	16	5
28	≥18	M	4.78	20	11	64	44	29	12	29	11
29	16–17	F	2.44	28	4	76	62	31	30	16	6
30	16–17	M	2.89	17	9	68	65	28	17	24	6
31	16–17	F	4.47	23	10	67	60	34	16	21	8
32	≥18	M	4.68	11	3	67	54	15	11	11	6

*M, male; F, Female*.

* *Age changed to ranges upon instruction from the journal*.

**Figure 2:**
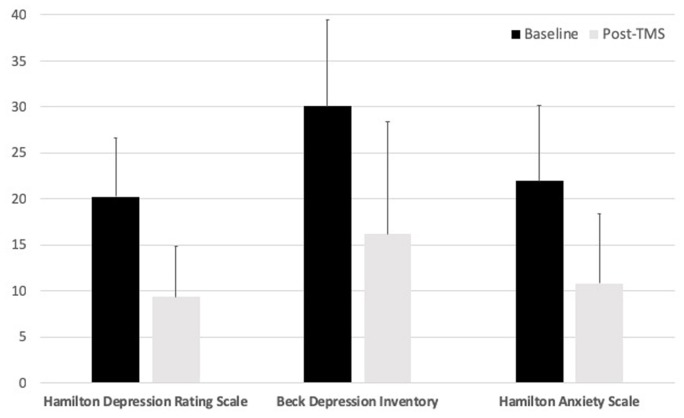
Summary of clinical outcomes. rTMS, repetitive transcranial magnetic stimulation.

From baseline to post-rTMS, Ham-D scores reduced significantly (*t* = 8.94, *df* = 31, *p* < 0.00001) (Figure 2). Based on our predefined definition of treatment response (≥ 50% reduction in Ham-D score), we observed 18 responders (56%) and 14 non-responders. Using a more liberal definition of response in keeping with ACNP guidelines (≥ 30% reduction), we observed 24 responders (75%) and 7 non-responders (19). At the post-rTMS assessment, 14 were rated by the Ham-D to be in the normal range, 13 as having mild depression, 1 as moderate, and 4 as severe. One participant moved up (mild to moderate). Furthermore, three mild, three moderate, two severe, and six very severe cases moved to the normal range. One mild case remained in the mild range. Six moderate, two severe, and four very severe cases moved to the mild range. Finally, four very severe cases moved to the severe range for depressive severity scores on the Ham-D. Fourteen subjects achieved remission (Ham-D score ≤ 7 post-rTMS), and 16 partial remission (Ham-D score ≤ 10 post-rTMS and ≥ 50% reduction in Ham-D score). There was no difference between medicated and un-medicated participants in terms of change in Ham-D (*t* = 0.29, *df* = 30, *p* = 0.78). CDRS and BDI scores reduced significantly with rTMS (*t* = 7.72, *df* = 31, *p* = 1.03 × ^−8^; *t* = 7.01, *df* = 31, *p* = 7.25 × ^−8^, respectively), as did Hamilton Anxiety Rating Scale scores (*t* = 8.23, *df* = 31, *p* < 0.00001).

In an exploratory manner, we looked at the effect of time (i.e., baseline, weeks 1, 2, and post-rTMS). A repeated measures ANOVA found a significant effect of time [Wilks' Lambda = 27.16, *F*_(3, 29)_ = 52.83, *p* < 0.001]. Follow up comparisons indicated that baseline depression scores were higher than each follow up time point (*p* < 0.001 each). Scores at the end of week 1 did not differ from week 2 (*p* = 0.52) but were higher than post-rTMS (*p* = 0.001). Scores at the end of week 2 did not differ from the end point (*p* = 0.15). Furthermore, we looked to see if there was a difference in outcome between age groups based on a median split (above and below 17.61 years). No difference was observed (*p* = 0.84).

No major adverse events were reported. Mild to moderate headaches were the most commonly reported side effect (31.25%), followed by mild neck pain (21.88%) (see Table 2). Neck pain was not reported after the use of an air-travel style pillow was started. Side effects were self-limiting and did not require medication. Tolerability scores improved over time for headache (Chi = 6.65, *p* = 0.01). Trial retention rate was 91% and compliance (completing all study events) was 99.28%.

**Table 2:** Incidence of headaches, neck pain, unpleasant tingling, nausea, and lightheadedness after 75 trains of high-frequency rTMS (120%RMT, 10 Hz) applied to the left DLPFC in adolescents with MDD (*n* = 32 participants, data from 96 safety and tolerability forms).

	**Week 1**	**Week 2**	**Week 3**
Headache	10 (6 mild, 4 moderate)	6 (5 mild, 1 moderate)	2 (1 mild, 1 moderate)
	31.25%	18.75%	6.25%
Neck pain	7 mild	4 mild	4 mild
	21.88%	12.50%	12.50%
Unpleasant tingling	6 (4 mild, 2 moderate)	4 mild	1 mild
	18.75%	12.50%	3.13%
Nausea	3 mild	1 mild	1 moderate
	9.38%	3.13%	3.13%
Lightheadedness	4 mild	2 mild	2 mild
	12.50%	6.25%	6.25%

## Discussion

To our knowledge, this is the largest open label trial of rTMS to date in youth with treatment resistant MDD. The results of this open label study indicate that rTMS may be clinically useful for treatment resistant depression in youth. However, in the absence of a sham control it is not possible to determine if improvement was due to rTMS alone. The side effects reported during this study were transient and typically mild. The most commonly reported side effect was a transient headache. However, because headaches are unusually common in the southern Alberta region (even in youth) it is impossible to directly correlate these headaches to the rTMS procedure (29). Neck pain was likely due to the need to remain still, or the comfort level of the TMS setup itself (chair/neck support). Pausing the procedure halfway through to stretch and move might help alleviate discomforts, and efforts should be made to examine this further. Our methodology also used a precision and personalized medicine approach with neuronavigation which may enhance the reliability of dosing rTMS from session to session. However, our findings must be considered in the context of limitations. The study design was an open and unmasked trial. The participants were allowed to either continue antidepressant medications or receive rTMS as monotherapy which presents potential confounds. We did not standardize brain state (i.e., all participants doing the same activity) during the TMS sessions. However, based on the present data the potential benefits of rTMS for MDD symptomology likely far outweigh the potential negative consequences of the procedure.

## Data Availability

All datasets generated for this study are included in the manuscript and/or the supplementary files.

## Author Contributions

All authors contributed to the drafting and revision of the manuscript, interpretation of the results, and data collection and analysis. FM, AK, TW, PC, NJ, and LL contributed to the study design.

### Conflict of Interest Statement

The authors declare that the research was conducted in the absence of any commercial or financial relationships that could be construed as a potential conflict of interest.
